# Combined functional genomic and metabolomic approaches identify new genes required for growth in human urine by multidrug-resistant *Escherichia coli* ST131

**DOI:** 10.1128/mbio.03388-23

**Published:** 2024-02-14

**Authors:** Minh-Duy Phan, Horst Joachim Schirra, Nguyen Thi Khanh Nhu, Kate M. Peters, Sohinee Sarkar, Luke P. Allsopp, Maud E. S. Achard, Ulrike Kappler, Mark A. Schembri

**Affiliations:** 1 Institute for Molecular Bioscience (IMB), The University of Queensland, Brisbane, Queensland, Australia; 2 School of Chemistry and Molecular Biosciences, The University of Queensland, Brisbane, Queensland, Australia; 3 Australian Infectious Diseases Research Centre, The University of Queensland, Brisbane, Queensland, Australia; 4 School of Environment and Science, Griffith University, Nathan, Queensland, Australia; 5 Griffith Institute for Drug Discovery, Griffith University, Nathan, Queensland, Australia; 6 Centre for Advanced Imaging, The University of Queensland, Brisbane, Queensland, Australia; National University of Singapore, Singapore, Singapore

**Keywords:** uropathogenic *Escherichia coli*, urinary tract infection, bacterial pathogenesis

## Abstract

Urinary tract infections (UTIs) are one of the most common bacterial infections in humans, with ~400 million cases across the globe each year. Uropathogenic *Escherichia coli* (UPEC) is the major cause of UTI and increasingly associated with antibiotic resistance. This scenario has been worsened by the emergence and spread of pandemic UPEC sequence type 131 (ST131), a multidrug-resistant clone associated with extraordinarily high rates of infection. Here, we employed transposon-directed insertion site sequencing in combination with metabolomic profiling to identify genes and biochemical pathways required for growth and survival of the UPEC ST131 reference strain EC958 in human urine (HU). We identified 24 genes required for growth in HU, which mapped to diverse pathways involving small peptide, amino acid and nucleotide metabolism, the stringent response pathway, and lipopolysaccharide biosynthesis. We also discovered a role for UPEC resistance to fluoride during growth in HU, most likely associated with fluoridation of drinking water. Complementary nuclear magnetic resonance (NMR)-based metabolomics identified changes in a range of HU metabolites following UPEC growth, the most pronounced being L-lactate, which was utilized as a carbon source via the L-lactate dehydrogenase LldD. Using a mouse UTI model with mixed competitive infection experiments, we demonstrated a role for nucleotide metabolism and the stringent response in UPEC colonization of the mouse bladder. Together, our application of two omics technologies combined with different infection-relevant settings has uncovered new factors required for UPEC growth in HU, thus enhancing our understanding of this pivotal step in the UPEC infection pathway.

**IMPORTANCE:**

Uropathogenic *Escherichia coli* (UPEC) cause ~80% of all urinary tract infections (UTIs), with increasing rates of antibiotic resistance presenting an urgent threat to effective treatment. To cause infection, UPEC must grow efficiently in human urine (HU), necessitating a need to understand mechanisms that promote its adaptation and survival in this nutrient-limited environment. Here, we used a combination of functional genomic and metabolomic techniques and identified roles for the metabolism of small peptides, amino acids, nucleotides, and L-lactate, as well as the stringent response pathway, lipopolysaccharide biosynthesis, and fluoride resistance, for UPEC growth in HU. We further demonstrated that pathways involving nucleotide metabolism and the stringent response are required for UPEC colonization of the mouse bladder. The UPEC genes and metabolic pathways identified in this study represent targets for the development of innovative therapeutics to prevent UPEC growth during human UTI, an urgent need given the rapidly rising rates of global antibiotic resistance.

## INTRODUCTION

Urinary tract infection (UTI) is one of the most common bacterial infections in humans, with ~400 million cases annually worldwide (1, 2). These infections can range from mild cystitis to more severe pyelonephritis and have high rates of recurrence. The primary causative agent of UTI is uropathogenic *Escherichia coli* (UPEC), which accounts for up to 80% of all cases (3). Of great concern are increasing rates of antibiotic resistance, highlighted by the emergence and spread of the pandemic UPEC sequence type 131 (ST131) clone associated with high rates of infection (4). Metadata analysis of 169 studies revealed that since the year 2000, ST131 is the most common clinical ST, with the prevalence of ST131 among multidrug-resistant (MDR) UPEC isolates as high as 62%, 3.6 times higher than the prevalence of ST131 among all *E. coli* irrespective of their resistance status (5). ST131 has also been reported as the second most common ST among carbapenemase-producing *E. coli* isolated in 36 countries between 2015 and 2017 (6), posing a significant challenge for antimicrobial treatment options. The *E. coli* strain EC958 is a prototypical ST131 strain that has been extensively studied over the past decade (7–11).

The ability of UPEC to grow efficiently in human urine (HU) is crucial for the establishment and progression of UTI. Indeed, UPEC grow rapidly in urine of patients with UTI during active infection (12). HU is a complex biological fluid and contains more than 400 metabolites that can be detected and quantified using a variety of analytical methods (13, 14). The primary sources of nutrients in HU include organic acids like hippuric acid and citric acid, as well as peptides, amino acids, nucleic acids, and various inorganic substances (13). Despite its limited nutritional value, previous studies that were based mostly on transcriptomic analyses have identified specific metabolic pathways and processes required for UPEC growth in HU, such as import of peptides, gluconeogenesis, the tricarboxylic acid cycle, iron acquisition, nitrate/nitrite metabolism, and nitric oxide protection (12, 15–19).

Transposon-directed insertion-site sequencing (TraDIS) and similar transposon-insertion sequencing methods such as TnSeq have been widely used as powerful functional genomics methods to understand the genetic basis of complex phenotypes associated with the ability of UPEC and other bacteria to thrive in infection-related conditions (9, 20–25). These approaches involve the generation of highly saturated transposon mutant libraries and their subsequent subjection to selection pressure imposed by a condition of interest, followed targeted deep sequencing to determine differences in the frequency of transposon insertions across the genome. The application of TraDIS in combination with other high-throughput methodologies offers the opportunity to understand bacterial adaptation and growth in HU in new light. In the case of HU, its diverse metabolite content presents the opportunity to use metabolomics to profile changes in metabolite content following bacterial growth. Indeed, recent advances in multi-omics analyses have seen metabolomics become more widely used in combination with technologies such as genomics, transcriptomics, proteomics, structural biology, and imaging to understand mechanisms and systems-level effects of metabolic pathways, biomarkers, and disease progression (26, 27).

In this study, we utilized a TraDIS functional genomic approach in combination with metabolomics to uncover genes and pathways required for efficient growth of ST131 UPEC in HU. TraDIS identified 24 genes required for growth in HU, which together affected at least 19 pathways including small peptide, amino acid and nucleotide metabolism, the stringent response, LPS biosynthesis, and fluoride resistance. In addition, metabolomic analysis identified L-lactate as a preferred carbon source for growth in HU, with its utilization by UPEC dependent on the LldD L-lactate dehydrogenase. We further demonstrated the role of nucleotide metabolism and the stringent response in UPEC colonization of the mouse bladder.

## RESULTS

### *E. coli* ST131 strains grow efficiently in human urine

**Fig 1:**
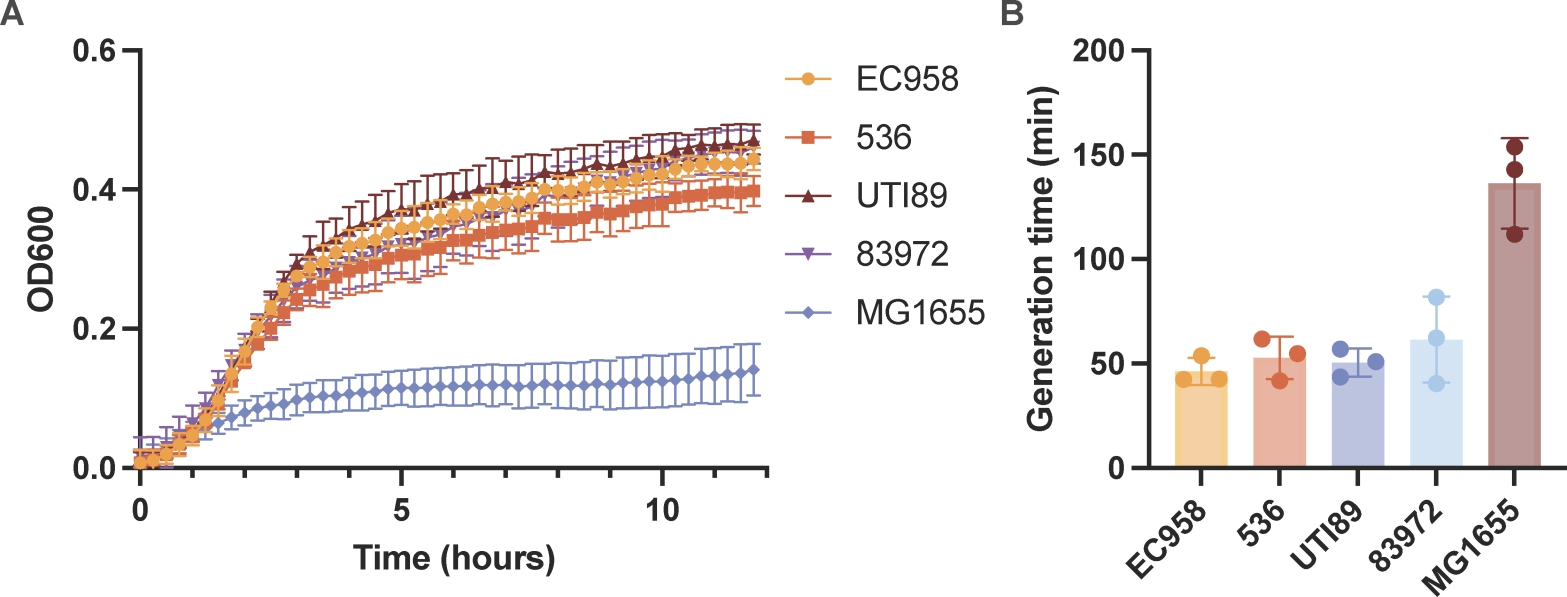
Growth characteristics of *E. coli* reference strains in HU. (A) Growth of UPEC (EC958, 536, and UTI89), ABU (83972), and K-12 (MG1655) strains in HU. Shown is the mean OD_600_ reading ± standard deviation. (**B)** Generation times of reference UPEC and ABU strains compared with MG1655. Generation times were calculated from the time range of 1–2 h. Shown is the mean and all data points from three independent experiments.

The ability to grow in HU is an essential criterion for UPEC to colonize the bladder. To investigate the growth characteristics of *E. coli* ST131 in HU, we compared the growth of the ST131 strain EC958 with the UPEC reference strains UTI89 and 536 and the asymptomatic bacteriuria (ABU) strain 83972. In this experiment, all strains grew similarly and significantly better than the K-12 strain MG1655 (Fig. 1A) with an average generation time of ~50 min for UPEC/ABU reference strains, as well as multiple additional ST131 strains (Fig. S1). This is in contrast to the K-12 reference strain MG1655, which had a generation time in HU of nearly 150 min (Fig. 1B). Our data confirmed that the *in vitro* growth characteristics of ST131 strains in HU are comparable to other well-characterized UPEC/ABU strains. Thus, the remainder of our study was performed using the ST131 strain EC958.

### Identification of EC958 genes required for growth in HU

**Fig 2:**
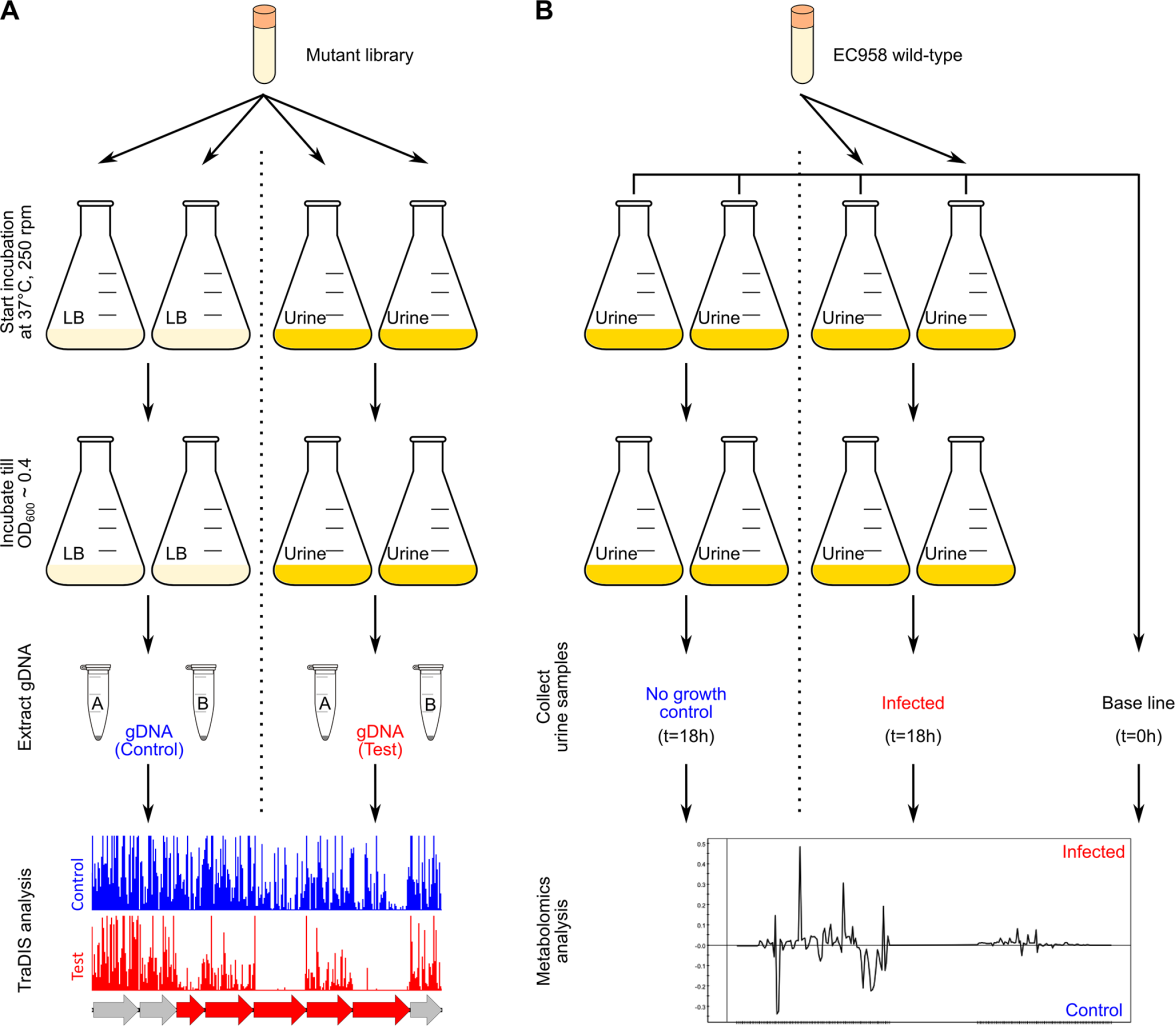
Experimental design of the combined TraDIS and metabolomics analyses. (A) Experimental design for TraDIS to identify genes required for growth in HU. (**B)** Experimental design for metabolomics analysis to identify changes in HU chemical components after EC958 growth.

We performed a genetic screen combining hyper-saturated miniTn*5*-transposon mutagenesis and TraDIS to identify genes required for EC958 growth in HU. This involved inoculation of 1 million mutants into pooled HU and incubation of the cultures at 37°C to an optical density (OD_600_) = 0.4 (Fig. 2A). During this procedure, bacteria containing mutations in genes required for growth in HU would be unable to grow and thus underrepresented or absent from the output pool. In parallel, the same number of mutants were inoculated into lysogeny broth (LB) and incubated under the same conditions to an OD_600_ = 0.4, thereby ensuring the libraries had gone through approximately the same number of generations in the HU selection and the LB broth control samples. Genomic DNA was extracted from the HU and LB broth samples and sequenced using our Illumina multiplexed TraDIS procedure as previously described (22) (Table S1).

Genes with a significant reduction in read counts in HU compared with LB broth were determined to be required for growth in HU. Using a stringent threshold (log_2_ fold change [logFC] of read counts ≤ −1 and adjusted *P* ≤ 0.001), we identified a total of 24 genes on the chromosome but no genes on the plasmid of EC958 (Table 1; Table S2; Fig. S2). Pathway analysis (EcoCyc [28]) revealed at least 19 metabolic pathways were affected by mutation of these 24 genes (Table S3). Six of these genes (*argGH*, *carB*, *metE*, and *serAC*) belong to pathways involved in amino acid biosynthesis and degradation, six genes (*birA*, *hns*, *lrp*, *oxyR*, *qseC*, and *plaR*) are regulators, and four genes (*guaAB*, *hpt*, and *purA*) belong to pathways involved in nucleotide biosynthesis and salvage. Other genes identified included *relA* (involved in the stringent response), *waaY* (involved in lipid A-core biosynthesis), and *crcB* (mediates resistance to fluoride).

**TABLE 1:** List of genes significantly involved in growth in human urine identified by TraDIS^*a*^

Locus tag	logFC	Name	Products (pathways)
**EC958_2814**	−4.186	guaA	GMP synthetase (glutamine aminotransferase) (guanosine ribonucleotides *de novo* biosynthesis/L-glutamine degradation I)
**EC958_4664**	−3.973	purA	Adenylosuccinate synthetase (adenosine ribonucleotides *de novo* biosynthesis)
**EC958_1510**	−3.124	hns	Global DNA-binding transcriptional dual regulator H-NS
**EC958_0743**	−3.046	crcB	Probable fluoride exporter [fluoride(cytosol) → fluoride(periplasmic space)]
**EC958_4753**	−2.787	holC	DNA polymerase III, chi subunit
**EC958_2815**	−2.480	guaB	IMP dehydrogenase (guanosine ribonucleotides *de novo* biosynthesis)
**EC958_3051**	−2.379	relA	(p)ppGpp synthetase I/GTP pyrophosphokinase (ppGpp biosynthesis)
EC958_2526	−2.276	yejM	Predicted hydrolase, inner membrane
**EC958_3422**	−1.575	qseC	Quorum sensing sensory histidine kinase (QseBC two-component signal transduction system)
**EC958_4446**	−1.482	argH	Argininosuccinate lyase (L-arginine biosynthesis I [via L-ornithine])
EC958_3573	−1.468	argG	Argininosuccinate synthetase (L-arginine biosynthesis I [via L-ornithine])
**EC958_4447**	−1.429	oxyR	Oxidative and nitrosative stress transcriptional regulator
EC958_3577	−1.325	folP	7,8-Dihydropteroate synthase (tetrahydrofolate biosynthesis)
EC958_3193	−1.273	serA	D-3-phosphoglycerate dehydrogenase (L-serine biosynthesis)
EC958_1001	−1.272	lrp	DNA-binding transcriptional dual regulator, leucine-binding
EC958_3119	−1.182	recD	Exonuclease V (RecBCD complex), alpha chain
EC958_1063	−1.119	serC	3-Phosphoserine/phosphohydroxythreonine aminotransferase (L-lysine biosynthesis I/L-serine biosynthesis/pyridoxal 5′-phosphate biosynthesis I)
**EC958_0166**	−1.083	carB	Carbamoyl-phosphate synthase large subunit (L-arginine biosynthesis I [via L-ornithine]/L-glutamine degradation I/UMP biosynthesis)
**EC958_4292**	−1.047	metE	5-Methyltetrahydropteroyltriglutamate- homocysteine S-methyltransferase (L-homoserine and L-methionine biosynthesis/L-methionine biosynthesis I/S-adenosyl-L-methionine cycle I)
EC958_5089	−1.032	birA	Bifunctional biotin-[acetylCoA carboxylase] holoenzyme synthetase/DNA-binding transcriptional repressor, bio-5′-AMP-binding (biotin-carboxyl carrier protein assembly)
**EC958_4032**	−1.029	waaY	Lipopolysaccharide core heptose (II) kinase (lipid A-core biosynthesis)
**EC958_0267**	−1.025	hpt	Hypoxanthine phosphoribosyltransferase (adenine and adenosine salvage III/guanine and guanosine salvage)
EC958_3981	−1.016	plaR	DNA-binding transcriptional repressor PlaR (negatively controls the expression of genes involved in the catabolism of the rare pentose L-lyxose)
EC958_4428	−1.002	metF	5,10-Methylenetetrahydrofolate reductase (N10-formyl-tetrahydrofolate biosynthesis)

^*a*^ Locus tags in bold indicate genes that were further characterized.

### Validation and comparative sequence analysis of genes required for growth in HU

**Fig 3:**
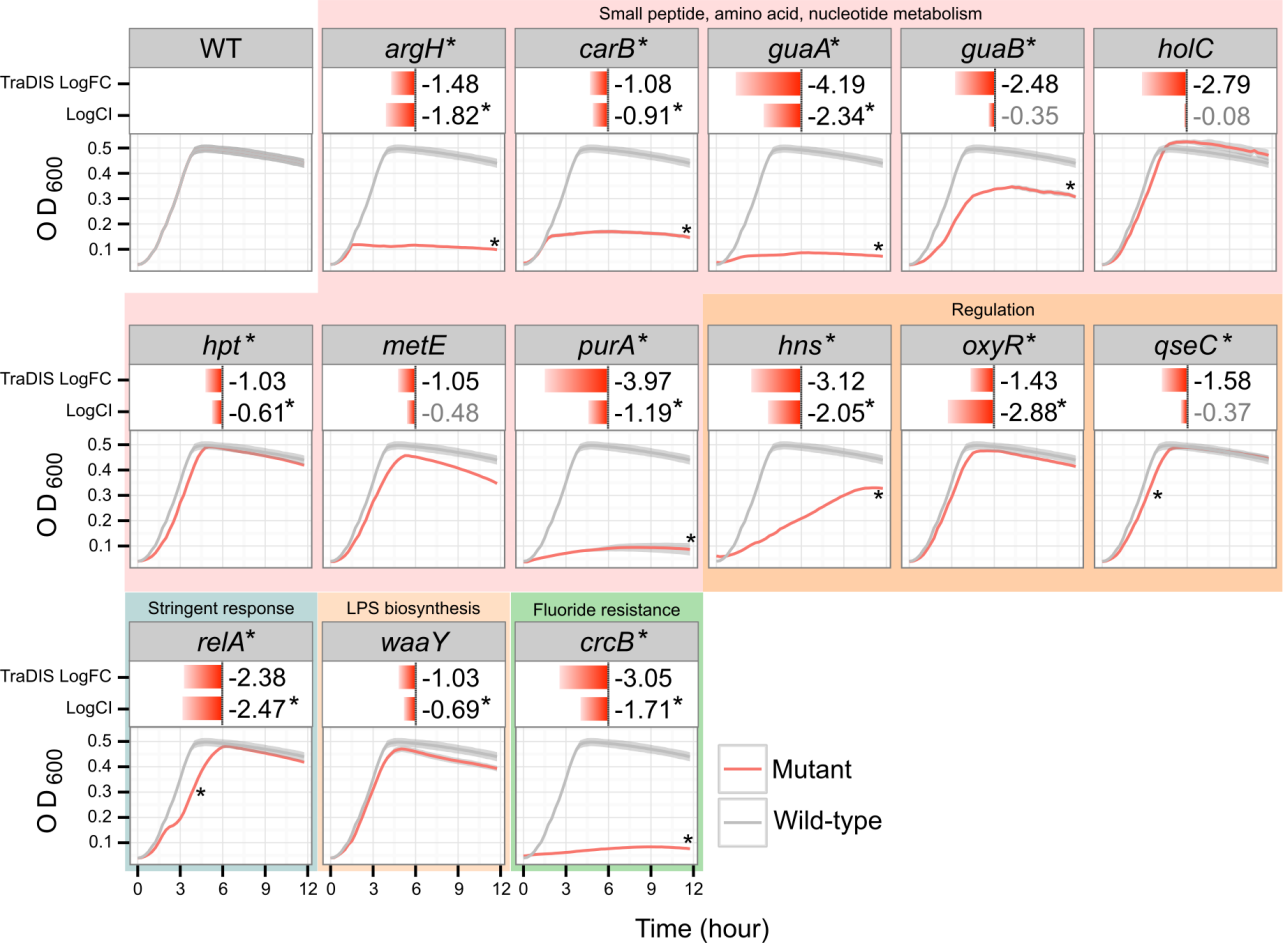
Growth characteristics of mutants identified by TraDIS in single and mixed competitive growth assays. Each panel presents the growth characteristics of a single mutant, including its growth (red) compared with the growth of WT EC958 (gray). logFC identified by TraDIS and the logCI calculated from competitive growth with WT EC958 in HU are shown above each growth curve. Mutants that exhibited a significant growth difference in HU compared with WT EC958 are indicated by an asterisk.

Out of 24 genes identified by TraDIS, we selected 14 genes for the generation of defined isogenic mutations; 8 genes involved in small peptide, amino acid, and nucleotide metabolism (*argH*, *metE*, *carB*, *guaA*, *guaB*, *hpt*, and *purA*); 3 genes involved in regulation (*hns*, *oxyR*, and *qseC*); and 1 gene each involved in replication (*holC*), stringent response (*relA*), LPS biosynthesis (*waaY*), and fluoride resistance (*crcB*) (Table 1; Fig. 3). These 14 mutants were used in single and mixed competitive assays to validate their role in growth in HU. Single-strain growth assays were performed in HU for 12 h, and the kinetic curves were compared with those of the wild-type (WT) EC958 strain. Using combined analysis criteria based on four parameters (maximum population size, growth rate, the time at which the population density reaches half maximum population size, and area under the curve), 9 out of 14 mutants displayed significantly reduced growth in HU (Fig. 3; Fig. S3). Mixed competitive growth assays were performed using an initial 50:50 ratio of each respective mutant in HU and WT EC958. In these experiments, all but four mutants (*guaB*, *holC*, *metE*, and *qseC*) showed a significant reduction in competitive fitness in HU (Fig. 3, log competitive index [logCI] value). Thus, in sum, 11 mutants exhibited growth defects in HU (indicated by an asterisk in Fig. 3).

We extended our analysis of the genes identified to be required for growth in HU by examining their prevalence and level of sequence conservation across a large data set comprising 10,000 *E. coli* genomes. The data set was generated by randomly selecting 100 genomes from the top 100 STs of *E. coli* from the public database EnteroBase as representative of major *E. coli* clones (referred to as the 100ST collection, previously used in reference 29). The sequences of the genes from EC958 were then compared (using BLASTN) with genomes in this 100ST collection. We observed that all genes are present in all other *E. coli* at 98%–100% prevalence except for *waaY* (reduced prevalence in all *E. coli*) and *plaR* in phylogroups D (present in 87% of genomes) and E (absent in these genomes). We also observed sequence variation for some genes at the phylogroup level. For example, the majority of *E. coli* strains in phylogroup B2 shared the same *argG* allele with EC958, but this allele was not found in any other phylogroups (Fig. S4). Together, this is suggestive of adaptive evolution where there may be functional differences for the proteins encoded by these genes.

### The stringent response is required for growth in HU

**Fig 4:**
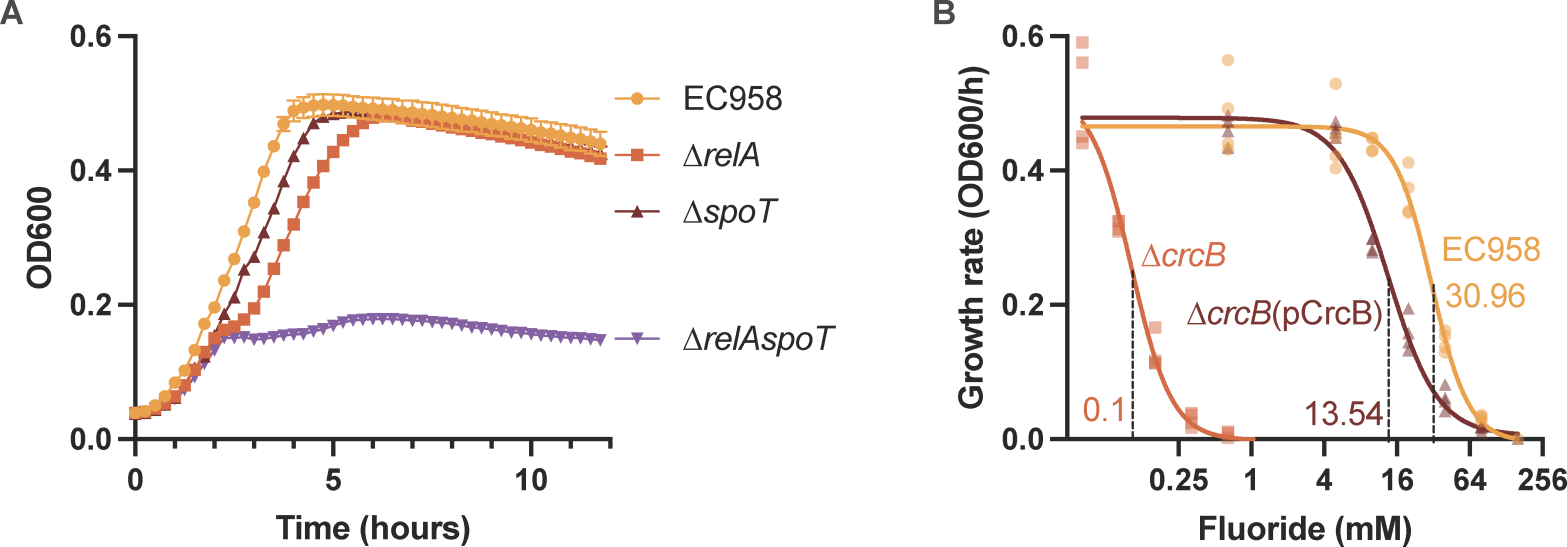
Roles of the stringent response and fluoride resistance for EC958 growth in HU. (A) Growth kinetics of EC958, EC958∆*relA*, EC958∆*spoT*, and EC958∆*relAspoT* in HU. Shown is the mean OD_600_ reading ± standard deviation from two replicates. (**B)** The effects of fluoride on growth rates (changes in OD_600_ per hour) of EC958, EC958∆*crcB*, and EC958∆*crcB*(pCrcB) in LB supplemented with NaF. The sigmoidal fits of the data are shown as lines, with IC50 indicated by the labels and dotted lines. All data points from four independent replicates are shown.

The stringent response is a conserved, global transcriptional adaptation to a range of nutritional and environmental stresses, including amino acid, iron, and fatty acid limitation (30, 31). The starvation response is mediated by the (p)ppGpp alarmone that can be produced by either the RelA or the SpoT enzymes and alters transcriptional patterns via interactions with RNA polymerase and accessory proteins (32). We identified *relA* as required in our TraDIS screen and confirmed that an EC958 ∆*relA* mutant (EC958∆*relA*) has a growth defect in HU (Fig. 3), consistent with the poor nutritional content of HU. Since RelA and SpoT act together to control the intracellular concentration of (p)ppGpp, we also generated a single deletion mutant of *spoT* (EC958∆*spoT*) and a double deletion of both *relA* and *spoT* (EC958∆*relAspoT*) and examined the role of these genes in EC958 growth in HU. In these experiments, the growth rate of EC958∆*relA* was significantly reduced compared with WT EC958; however, this mutant eventually reached an equivalent cell density after 12 h of growth (Fig. 4A). In contrast, EC958∆*spoT* exhibited similar growth kinetics to WT EC958 but reached a slightly decreased final cell density at stationary phase (9–12 h; Fig. 4A). The EC958∆*relAspoT* double mutant was significantly attenuated (Fig. 4A), confirming the essential role of stringent response for growth in HU.

### Fluoride resistance is required for growth in HU

Our TraDIS screen identified an essential role for the *crcB* gene during growth in HU. The *crcB* gene encodes a fluoride efflux pump, the expression of which is controlled by a fluoride-sensing riboswitch (33). We measured the concentration of fluoride in the HU sample used in our TraDIS experiment and determined it to be 0.074 mM (1.4 mg/L; Table S4). To characterize the effect of fluoride on EC958, we assayed the growth kinetics of WT EC958, EC958∆*crcB* mutant, and the EC958∆*crcB*(pCrcB)-complemented strain in LB supplemented with various concentrations of fluoride (Fig. 4B). Sigmoid fits of growth rates versus fluoride concentrations indicated that the IC50 of EC958∆*crcB* was significantly lower than that of WT EC958 (0.1 mM versus 30.96 mM). Complementation partially restored the IC50 to near WT level (13.54 mM). Taken together, our data demonstrate that resistance to fluoride is required for the growth of EC958 in HU due to the presence of fluoride.

### Metabolomic analysis of HU

**Fig 5:**
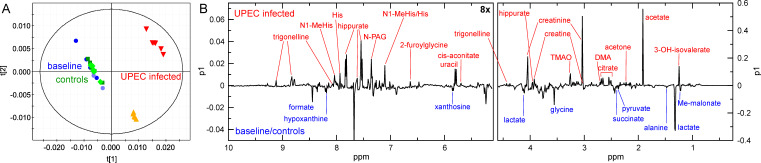
Metabolomic characterization of metabolite changes in EC958-infected HU samples compared with control samples.(**A)** PCA scores plot of fresh sterile HU (replicate 1, blue circles; replicate 2, light-blue circles), sterile HU incubated for 18 h (replicate 1, dark-green squares; replicate 2, green diamonds), and (**C**) HU incubated with EC958 for 18 h (replicate 1, orange triangles; replicate 2, red inverted triangles). The infected samples separated clearly from all control samples. (**B)** PC1 loadings line plot, identifying the metabolites that are increased (positive loadings coefficients, annotated in red) or decreased (negative loadings coefficients, annotated in blue) in infected compared with control HU samples. The vertical scale in the left-hand side of panel B is increased eightfold to aid visibility. The scores plot indicates how similar the metabolic profiles of the samples are to each other. Each data point represents one NMR spectrum (sample); clustering of data points in the scores plot is indicative of samples with a similar metabolite composition. The loadings plot illustrates the metabolites responsible for the variation within the samples observed in the respective position in the corresponding scores plot.

To complement our TraDIS analysis, we also characterized the metabolite changes in HU resulting from EC958 growth using NMR-based metabolomics (Fig. 2B). One-dimensional ^1^H NMR spectra were acquired for (i) fresh sterile HU (baseline), (ii) sterile HU incubated for 18 h (negative control), and (iii) HU incubated with EC958 for 18 h and analyzed using multivariate statistical analysis (MVSA). The principal component analysis (PCA) scores plot of the HU spectra demonstrated that baseline HU (blue) and negative control HU (green) samples were similar, whereas EC958-infected HU separated from the controls in principal component (PC) 1 (Fig. 5A). Although the two infected biological replicates differed from each other (vertical separation in PC2), this was primarily due to higher levels of acetate and lower levels of creatinine in the second biological replicate (red) compared with the first (orange), rather than differences in the types of metabolites present.

The loadings plot identified the metabolites that differed between infected and control HU samples. Here, levels of acetate, acetone, cis-aconitate, citrate, creatine, creatinine, dimethylamine, 2-furoylglycine, hippurate, histidine, 3-hydroxyisovalerate, N1-methyl-histidine, N-phenylacetylglycine, trimethylamine-N-oxide, trigonelline, and uracil increased, while alanine, formate, glycine, lactate, hypoxanthine, methylmalonate, pyruvate, succinate, and xanthosine concentrations decreased in the EC958-infected HU samples compared with the controls (Fig. 5B; Table S5). While it is interesting to note that both TCA cycle precursors and intermediates as well as nucleobases were among the metabolites consumed by EC958, the most pronounced metabolome changes were the increase in acetate in the infected HU samples and the decrease in lactate concentration, suggesting that lactate was used as a carbon source to support EC958 growth. Pathway analysis indicated that the major metabolic pathways affected were glycine, serine, and threonine metabolism; pyruvate metabolism; glyoxylate and dicarboxylate metabolism; and the TCA cycle (Fig. S5).

### L-Lactate is used as a carbon source in urine

The metabolomic analysis of HU provided strong evidence that lactate acts as a carbon source for EC958 growth. We determined that the lactate in the batch of HU used for the TraDIS screen was predominantly the L-lactate stereo-isomer that is more commonly present in the human body. Growth of EC958 in this batch of HU led to a decrease of L-lactate concentrations from 34.5 mg/L to 6.5 mg/L following 18 h of growth. In contrast, the amount of D-lactate did not change significantly (8.1 mg/L in the baseline HU sample compared with 5.6 mg/L in the HU sample incubated with EC958 for 18 h) (Table S4).

**Fig 6:**
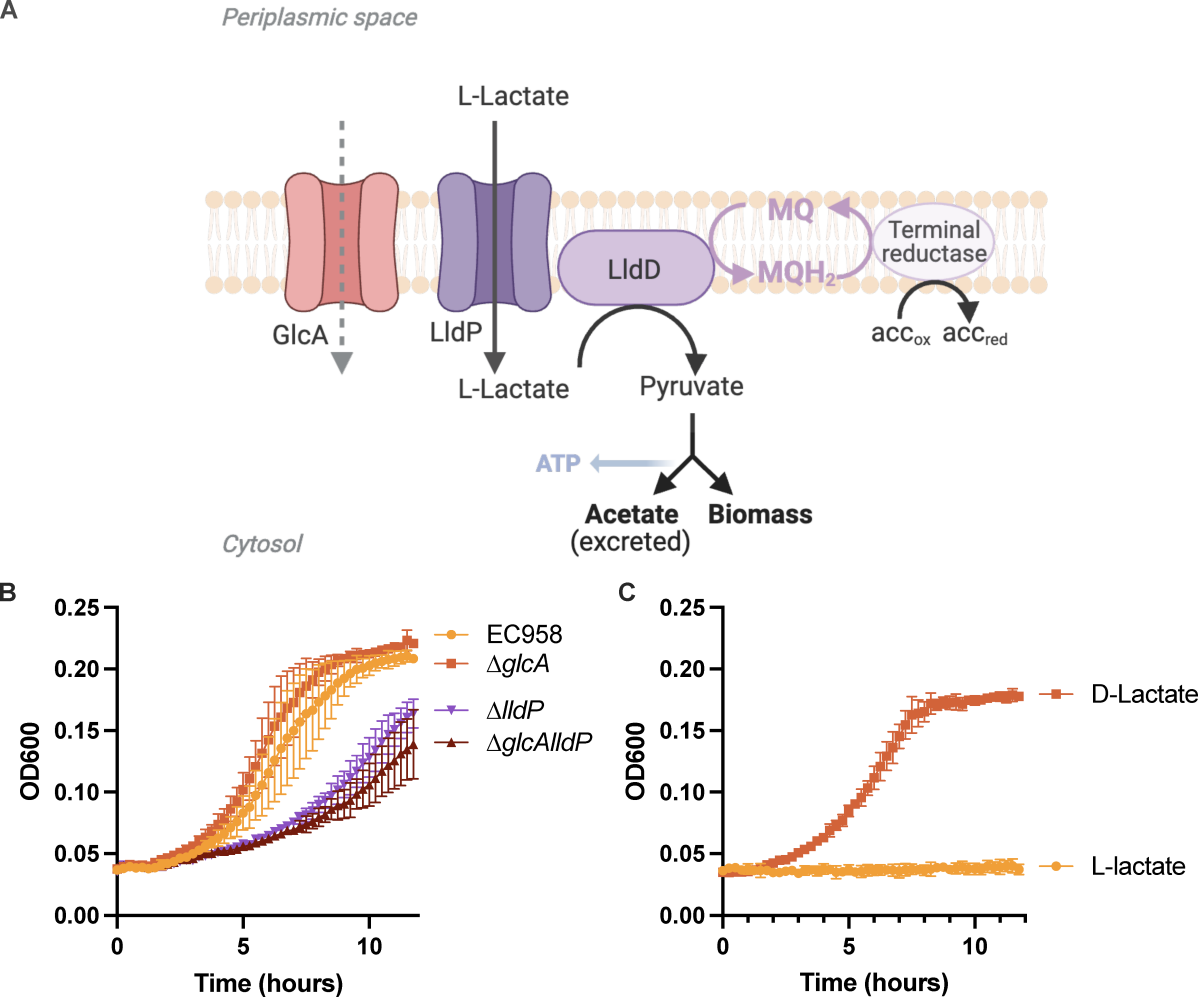
. Roles of lactate permeases and lactate dehydrogenase in the metabolism of L-lactate as a sole carbon source. (A) L-Lactate from the environment is taken up via L-lactate permease and then converted to pyruvate by L-lactate dehydrogenase. Pyruvate can then either be assimilated into biomass or converted to acetyl coenzyme A and subsequently to acetate while producing ATP. (**B)** Growth kinetics of lactate permease mutants in M9 media containing 0.2% L-lactate. (**C)** Growth kinetics of the L-lactate dehydrogenase mutant in M9 media containing either 0.2% L-lactate or 0.2% D-lactate.

L-Lactate can act as a substrate in bacterial carbon metabolism, where L-lactate can be taken up from the environment and converted to pyruvate by an L-lactate dehydrogenase, such as the *E. coli* LldD enzyme. Pyruvate can then either be assimilated into biomass or converted to acetyl-coenzyme A and subsequently to acetate while producing ATP (Fig. 6A). Acetate can also be produced directly from pyruvate via the PoxB pyruvate oxidase, but this reaction does not lead to ATP production.

In order to investigate the ability of EC958 to take up L-lactate, we generated targeted mutations in the *lldP* and *glcA* lactate permease genes located on the EC958 chromosome. We constructed both single (EC958∆*lldP* and EC958∆*glcA*) and double (EC958∆*glcAlldP*) mutants and grew these strains (as well as WT EC958) in minimal M9 media containing 0.2% L-lactate as a sole carbon source (Fig. 6B). Only the ∆*lldP* mutants (EC958∆*lldP* and EC958∆*glcAlldP*) exhibited attenuated growth, demonstrating that LldP is the main lactate permease in EC958 and that GlcA did not contribute to the uptake of lactate. We also examined the role of the *lldD* L-lactate dehydrogenase, which converts L-lactate to pyruvate and belongs to the same operon as *lldP* and the regulator *lldR*. Mutation of the *lldD* gene in EC958 (EC958∆*lldD*) completely abrogated growth on L-lactate but had no effect on growth with D-lactate (Fig. 6C). Thus, LldD is the only L-lactate dehydrogenase in EC958, and loss of this enzyme abolishes the ability of EC958 to utilize L-lactate as a carbon source.

### Genes required for nucleotide metabolism and the stringent response also contribute to colonization of the mouse bladder

**Fig 7:**
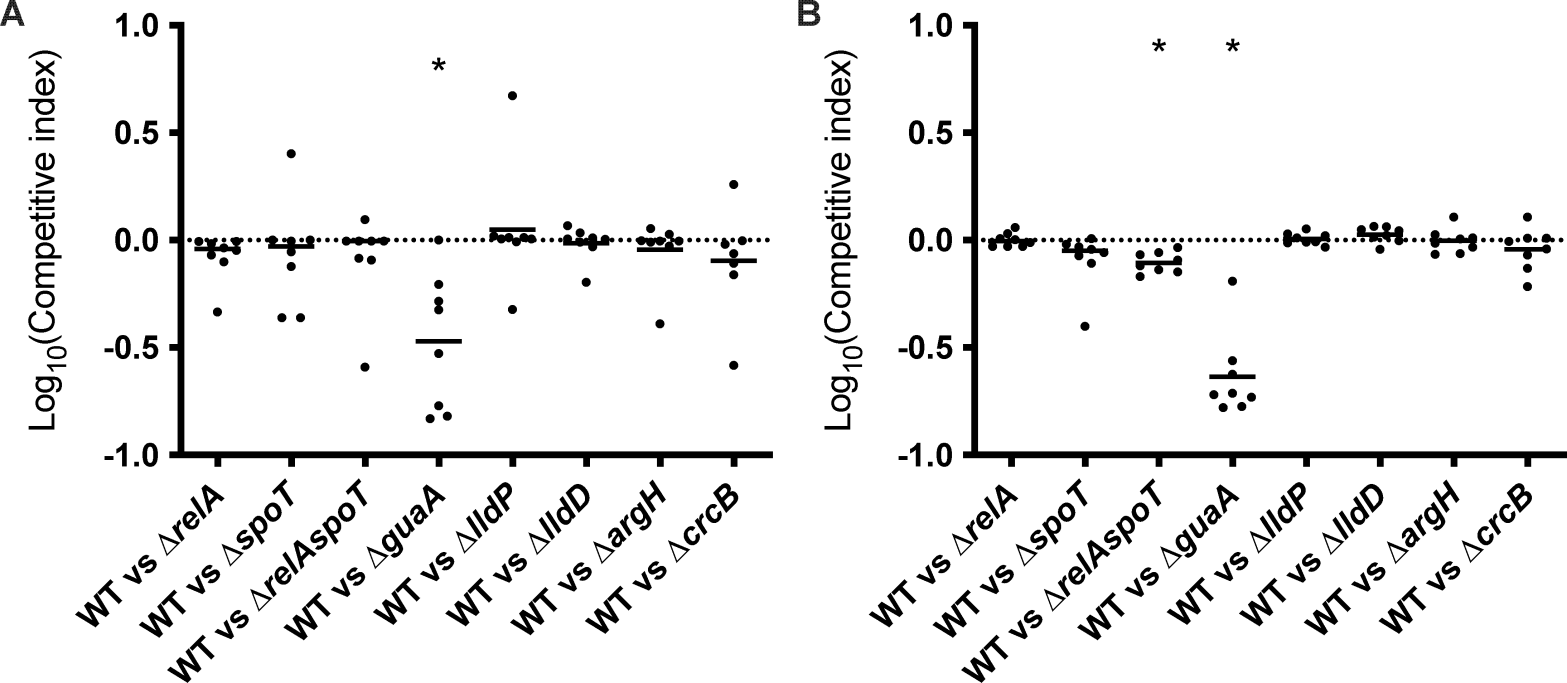
Mixed competitive infection in the mouse UTI model demonstrates a requirement for nucleotide metabolism and stringent response. Data are shown for the colonization of (**A**) urine and (**B**) bladder. Each symbol represents data from an individual mouse at 24 h post infection. Logarithmic values of competitive indices are plotted on the y axis; horizontal bars represent median values. A Log_10_ competitive index below 0 (shown by the dotted line) indicates that the mutant was at a competitive disadvantage. Statistical significance was determined by the two-tailed Wilcoxon’s signed rank test (*P < 0.05).

To investigate the significance of our findings *in vivo*, we selected mutant strains representing the major gene categories identified in our TraDIS and metabolomic screens and tested their capacity to colonize the mouse bladder using mixed competitive infection assays. Each mutant was co-infected with WT EC958 at a 50:50 ratio, and the log competitive index was determined for colonization of urine and the bladder by each strain pair at 24 h post infection. In mouse urine, only the ∆*guaA* mutant showed a significant reduction in colonization, indicating a requirement for *de novo* guanine production during growth in HU (Fig. 7A). In the mouse bladder, both the ∆*guaA* mutant and the ∆*relAspoT* double mutant were significantly outcompeted by WT EC958 (Fig. 7B).

## DISCUSSION

High morbidity and mortality associated with infections caused by multidrug-resistant pathogens have created an urgent need to understand disease pathogenesis and apply this knowledge to develop new therapeutics (2). This is particularly highlighted by ST131, which emerged recently and has rapidly become the most globally dominant ST among MDR UPEC (5). By employing the ST131 reference strain EC958 to investigate mechanisms for growth and survival in HU, we have identified critical metabolic, resistance, and adaptation pathways required for infection, many of which also serve as potential therapeutic targets.

Growth kinetic analyses demonstrated that the ST131 strain EC958 exhibited a similar growth profile in HU to other ST131 strains as well as UPEC and ABU reference strains (Fig. 1; Fig. S1). This is consistent with previous work that reported UPEC doubling times ranging from 45 min to 60 min at exponential phase in HU (34). Interestingly, even faster UPEC growth rates have been reported during active human UTI (12, 35) and in bladder epithelial cells of experimentally infected mice (12, 36). Together, despite extensive UPEC strain variation and the analysis of UPEC growth in *in vitro* and *in vivo* settings, the fact that ST131 growth occurs at a rate similar to non-ST131 UPEC suggests that the genes and pathways identified in this study are likely broadly relevant to all UPEC.

Our genetic screen employing TraDIS identified 24 genes on the EC958 chromosome, encompassing at least 19 pathways, to be essential for growth in HU (Table 1; Table S3). Small peptide, amino acid, and nucleotide metabolism have previously been associated with growth in HU (15–18) and were also identified in our study. It is noteworthy that our TraDIS approach only identified individual genes in these pathways. This is not fully unexpected, as metabolic pathways are highly interconnected, and in many cases, redundancies in genes encoding specific enzymes may mask effects of individual mutations. Additionally, the low detection rate of other pathway genes may reflect a limitation in the screen, which enriched for mutants able to grow in HU but in most cases may not have fully eliminated mutants that were unable to grow or able to grow at reduced rates. Nevertheless, our TraDIS analysis still identified a set of significant genes required for growth in HU, all of which have previously been shown to be highly expressed in UPEC strains examined directly from the urine of patients suffering acute UTI (Fig. S6A) (18). We also showed that many of these genes exhibit phylogroup-level allelic variation, consistent with a previous report that showed the *argI* variant in UPEC strains from phylogroup B2 has diverged from allelic variants in other *E. coli* phylogroups and is linked with persistent infection of the mouse bladder (37). The identification of the *qseC* gene, which encodes a sensor kinase that responds to various signals and phosphorylates the QseB response regulator (38, 39), is also in line with its role in the regulation of nucleotide, amino acid, and carbon metabolism (40). Finally, we were able to validate a role for 11 genes identified in our screen based on targeted mutation and subsequent phenotypic analysis. No genes on the plasmid pEC958 were identified as required for growth in HU. However, this is not surprising since this plasmid belongs to the replicon type F2:A1:B- that is most common in clade C2 of ST131 and largely carries genes conferring antibiotic resistance (11, 41, 42).

A role for the stringent response pathway has not been described previously for UPEC growth in HU or bladder colonization. The stringent response is regulated by the effector alarmone (p)ppGpp, the synthesis of which is controlled by the enzymes RelA and SpoT in *E. coli*. RelA-mediated synthesis of ppGpp is triggered by uncharged tRNA in the ribosomal A-site during amino acid starvation (43) or by NtrC-mediated activation of *relA* transcription during nitrogen starvation (44, 45), whereas SpoT is responsible for the synthesis of (p)ppGpp in response to other stresses and nutrient limitation and this enzyme is also capable of hydrolyzing (p)ppGpp (46–48). The stringent response thus plays a crucial role in growth adaptation during amino acid downshift and coordination of transcription-translation during carbon downshift (49). We identified *relA* in our TraDIS screen and thus generated a set of EC958 isogenic ∆*relA*, ∆*spoT*, and ∆*relAspoT* mutants. EC958∆*relA* exhibited a slower growth rate in HU, while growth of EC958∆*relAspoT* was severely attenuated (Fig. 4A). This could indicate EC958 growing in HU was undergoing amino acid starvation that may have triggered a RelA-mediated stringent response, which matches the known, low concentrations of amino acids in HU. Our data indicated this was mostly genes involved in the biosynthesis of arginine and serine. The stringent response plays a role during infection in multiple pathogens, including *E. coli*, by affecting adhesion, invasion, immune evasion, dissemination, biofilm formation and chronic infection (32). Indeed, our data showed a reduction in competitive fitness of EC958∆*relAspoT* compared with the EC958 WT during bladder colonization in the mouse UTI model (Fig. 7B). We also identified a role for *guaA* in bladder colonization (Fig. 7B). The *guaA* gene encodes a GMP synthetase that catalyzes the glutamine-dependent synthesis of GMP from XMP in the guanosine ribonucleotide *de novo* biosynthesis pathway (50). Since the (p)ppGpp alarmone also inhibits GTP synthesis (51), the mutation of *guaA* would reduce the pool of intracellular guanine and therefore the GTP concentration. This would not only affect cellular processes dependent on guanine availability but would create an effect that mimics GTP depletion during the stringent response, consistent with a previously identified role for the *guaB* IMP dehydrogenase (52). Interestingly, inhibitors of the stringent response pathway have been developed, either by blocking the activity of RelA and SpoT (53, 54) or by inhibiting the function of the (p)ppGpp alarmone (55, 56). Testing the efficacy of these small molecule inhibitors for the treatment of UTI would be an interesting avenue for future research.

Fluoride resistance is common to many bacteria, which use fluoride sensing RNAs to control the expression of a fluoride efflux pump that prevents the accumulation of this toxic anion in the cytoplasm (33). Our TraDIS experiment identified the *crcB* gene, encoding a fluoride-specific ion channel belonging to the Fluc-family (57), as required for growth in HU. Indeed, this finding was consistent with the presence of fluoride in our HU samples, which we determined to be 0.074 mM (1.4 mg/L; Table S4), consistent with the recommended level of fluoride in Queensland drinking water (0.6–0.8 mg/L) (58). We used a defined *crcB* mutant and complemented strain to confirm this observation, revealing that EC958∆*crcB* exhibited an IC50 of 0.1 mM fluoride in LB media, and this was partially restored by complementation to 13.54 mM, compared with WT at 30.96 mM (Fig. 4B). The concentration of fluoride in HU depends on the total dietary intake, of which drinking water is the largest source, but other sources can include dental products, supplements, and dietary products that contain naturally occurring fluoride. The requirement for a functional CrcB transporter for UPEC growth in HU raises the intriguing possibility that resistance to fluoride may contribute to the spread of specific UPEC clones in regions where fluoridation of drinking water supplies is performed.

Metabolomic profiling performed on HU samples to complement the TraDIS analysis revealed that multiple amino acids (e.g., alanine, glycine), nucleobases and nucleosides (e.g., hypoxanthine and xanthosine), and carboxylic acids (e.g., formate, lactate, pyruvate, succinate, and methylmalonate) were decreased after EC958 growth, suggesting the ability of EC958 to utilize these molecules as substrates for growth (Fig. 5; Table S5). The largest changes were the reduction of lactate and the increase in acetate concentrations, suggesting EC958 may have consumed lactate in HU to produce acetate and energy. L-Lactate is more reduced than biomass, and we propose that the reduced quinones produced during conversion of L-lactate to pyruvate would be re-oxidized via either the bd-oxidase or other, alternative terminal reductases as has been documented in other bacteria (59, 60) (Fig. 6A). An important aspect for carbon source utilization is the ability to take it up from the environment, and *E. coli* strains harbor two lactate permeases, LldP and GlcA (61). Using isogenic mutants derived from EC958, we demonstrated a role for LldP, but not GlcA, as the sole transporter of L-lactate. While the importance of L-lactate utilization by UPEC during UTI has not been investigated in detail, previous transcriptomic (RNA-seq) data support a role for this metabolic pathway. First, the expression of *lldR* (regulator of the *lldDRP* operon) and *lldP* (permease) by UPEC strains analyzed directly from the urine of patients suffering acute UTI was significantly higher than the average expression level of all genes in all samples (Fig. S6A) (18). Second, in another study that compared the transcriptome of five UPEC isolates in LB media, HU, and HU during active UTI, *lldD* was expressed at a higher level in HU compared with LB and up to ~10-fold higher during active UTI compared with HU (Fig. S6B) (17). Taken together, our data indicate that L-lactate should be considered as one of many substrates used by UPEC to support growth in the nutritionally poor environment of the urinary tract.

In summary, using a combination of functional genomic and metabolomic methods, we demonstrated a role for nucleotide biosynthesis, L-lactate utilization, the stringent response pathway, and fluoride resistance in promoting UPEC growth in HU. Additionally, we also demonstrated the significance of nucleotide metabolism and the stringent response in facilitating UPEC colonization of the mouse bladder. These UPEC genes and metabolic pathways represent potential targets for developing new therapeutics aimed at inhibiting UPEC growth during human UTI, a critical priority given the rapid rise of global antibiotic resistance.

## MATERIALS AND METHODS

### Bacterial strains and growth conditions

This study used *E. coli* EC958 which was isolated from the urine of a patient presenting with community UTI in the northwest region of England and is a representative of *E. coli* ST131 clade C2 (7, 8, 62). Other *E. coli* ST131 strains were from a global collection of ST131 (4). Bacterial strains were routinely cultured at 37°C on solid or in liquid LB medium supplemented with the appropriate antibiotics (chloramphenicol 30 µg/mL or gentamicin 20 µg/mL) unless indicated otherwise.

### Transposon mutant library screen

The EC958 mini-Tn*5* mutant library was generated previously (22). Fresh HU was collected from three healthy women who did not have any antibiotic treatment in the last 3 months. The HU was pooled in equal volume, centrifuged at maximum speed to remove cell debris, and then filter sterilized using a 0.22-µm Corning bottle-top vacuum filter system. Freshly pooled HU was used for the TraDIS experiments, and the excess was frozen at −80°C in small aliquots for metabolomic profiling, other downstream analysis, and phenotypic assays.

Approximately 2 × 10^8^ viable mutants were incubated in 100 mL of pooled HU (Test) and in 100 mL LB (Control) at 37°C with 250 rpm shaking (starting inoculum at ~2 × 10^6^ CFU/mL). The Test samples were incubated for 18 h (~3.5 × 10^8^ CFU/mL) while the Control samples were incubated for 3 h (~1.0 × 10^8^ CFU/mL) to match the number of generations (estimated at the time of collection by OD_600_). Both Control and Test experiments were performed in duplicate. Bacterial cells were collected, and genomic DNA was extracted from 5 mL of each culture using Qiagen 100-G genomic tips. Multiplex TraDIS was performed as previously described (22).

### TraDIS analysis

Sequence reads in FASTQ format were split into samples using the Tn5-specific tags and index sequences, trimmed for low-quality bases, and mapped to the EC958 genome (GenBank: HG941718) as previously described (22). Genes required for growth in HU were identified by comparing the differences in read abundance for each gene between the LB control and HU test samples using the Bioconductor package edgeR (version 3.4.2) (63). To match the number of generations in the LB control and HU test, the DNA from LB and HU samples was extracted at different growth phases, leading to read count bias caused by replication forks in the mid-log phase LB samples compared with the late stationary phase HU samples. To correct for replication fork bias, the generalized additive model (mgcv package version 1.8) was used to fit a regression smooth curve of read counts against genomic locations. This regression curve was then used to correct for count bias. The corrected read counts from two biological replicates of each treatment were loaded into edgeR using the R environment (version 3.03) and analyzed as previously described (22). Stringent criteria of log fold change (logFC) ≤ −1 and false discovery rate ≤ 0.001 were used to define a list of the most significant genes for further investigation by phenotypic analysis. Genes identified by TraDIS were mapped to pathways using the EcoCyc Omics Viewer (64).

### Molecular and bioinformatic methods

Chromosomal DNA purification, PCR, and DNA sequencing of PCR products were performed as previously described (65). Defined mutations were made using the λ-Red recombinase method with some modifications as previously described (22, 66, 67). Complementation was performed by cloning the gene of interest into a gentamicin-resistant derivative of pSU2718 (68) at appropriate cut sites. The genome assemblies of 100 randomly selected isolates from each of the 100 most common STs in EnteroBase (69) were downloaded on 18 December 2020 as described previously (29).

### Bacterial growth kinetics

Bacterial growth kinetics were measured as changes in OD_600_ over time using the FLUOstar OPTIMA Microplate Reader (BMG Labtech) as previously described (29). Briefly, the cultures were started at initial inoculum of OD_600_ = 0.05 in 200 µL per well of a flat-bottom 96-well plate, sealed with Breathe-Easy sealing membrane (Diversified Biotech), and incubated at 37°C with shaking inside the microplate reader. Optical measurements were recorded every 15 min for 12 h; all experiments were performed in at least duplicate. Parameters of the growth curves were estimated by the growthcurver (v0.3.1) package, and the *t*-test comparing mutants and WT was performed in R (v4.1.3) using the tidyverse (v1.3.1) package.

### Mixed competition assays

Competition assays were performed using similar conditions as described for bacterial growth kinetics. A 50:50 mixture of WT (EC958∆*lac*) and mutant strains was used. Viable counts were determined at *t* = 0 h and *t* = 18 h by plating on MacConkey lactose agar, which allowed the differentiation of EC958∆*lac* (non-lactose fermenter) and the mutant strains. The log competitive index was calculated using the following formula: log[(CFU_t18-MT_/CFU _t18-WT_)/(CFU_t0-MT_/CFU_t0-WT_)]. Our previous work has demonstrated the EC958∆*lac* mutant does not display a difference in colonization of the mouse bladder compared with WT EC958 in our experimental model (22).

### Metabolomic analysis

#### Design

The following urine samples were analyzed with NMR-based metabolomics: (i) sterile urine (baseline), (ii) sterile urine incubated for 18 h (negative control), and (iii) urine incubated with EC958 for 18 h at 37°C with 250 rpm shaking. Group (i) used *n* = 2 biological replicates and *n* = 5 technical replicates, while groups (ii) and (iii) used *n* = 2 biological replicates and *n* = 6 technical replicates, yielding a total of 34 samples.

#### Sample preparation

Five percent (wt/vol) sodium azide solution was added to sterile-filtered urine samples to a final concentration of 0.05% (wt/vol). For each NMR sample, aliquots of 450 µL urine were mixed with 50 µL 1.5 M sodium/potassium phosphate buffer, pH 7.4, and 50 µL of a solution containing 1 mM 2,2-dimethyl-2-sila-3,3,4,4,5,5-hexadeuteropentane-5-sulfonic acid (DSS) as a chemical shift standard and 1 mM 1,1-difluoro-1-trimethylsilanyl methyl phosphonic acid (DFTMP) as internal pH standard, both dissolved in D_2_O. Each 550 µL NMR sample was transferred into a 5-mm NMR sample tube.

#### NMR spectroscopy

^1^H NMR spectra were acquired at 298 K on a Bruker AV500 NMR spectrometer operating at a ^1^H frequency of 500.13 MHz, equipped with a 5-mm self-shielded z-gradient triple resonance probe, and a B-ACS 60 sample changer. 1D NOESY spectra were acquired in automation mode with the standard noesypr1d pulse sequence [(RD)−90°-t_1_-90°-τ_m_-90°-acq] (Bruker Biospin pulse program library). The water signal was suppressed by continuous wave irradiation during both the relaxation delay of 2.3 s and the mixing time (τ_m_) of 100 ms. With a spectral width of 14.0 ppm , 32,768 data points were collected into 128 transients and 8 dummy scans. All spectra were processed with TOPSPIN version 1.3 (Bruker Biospin, Rheinstetten, Germany). The free induction decays were multiplied by an exponential window function corresponding to a 0.1-Hz line broadening factor before Fourier Transformation. The acquired spectra were manually phased, segment-wise baseline corrected with polynomial functions, and referenced against the DSS signal at δ = 0 ppm.

#### Data handling

The processed NMR spectra were exported into MATLAB and aligned using the icoshift algorithm (70). Using an in-house MATLAB script, the aligned spectra were data reduced into regions of 0.01-ppm width (buckets) in the range of 10–0.25 ppm, excluding the region around the water signal between 5.12 and 4.62 ppm. Each bucketed spectrum was normalized to the total signal intensity.

#### MVSA

The bucketed spectra were analyzed with center-scaled PCA in the software package SIMCA-P+ 12.0 (Umetrics AB, Umeå, Sweden). After initial PCA (data not shown), sample outliers were removed, yielding a stable PCA model with *n* = 31 samples, *k* = 925 variables (buckets), *A* = 6 principal components, *R*^2^*X* = 0.964, and *Q*^2^ = 0.747.

#### Metabolite identification and univariate statistical analysis

The PCA loadings plot shows urinary metabolites affected by UPEC growth, which were identified in Chenomx NMR Suite version 7.7 (Chenomx Inc., Edmonton, Canada). Subsequently, the normalized relative concentrations of these metabolites were analyzed with univariate analysis. Student’s *t*-test was performed on the normalized relative concentrations to evaluate whether they were significantly different (*P* < 0.05 or *P* < 0.001) between the UPEC-infected and baseline control samples. The Bonferroni correction was used to adjust the raw *P*-values for multiple testing.

#### Metabolic pathway analysis

Metabolic pathway analysis, which combines pathway enrichment analysis and topology analysis, was performed in MetaboAnalyst 4.0 (71, 72). Files containing the normalized relative metabolite concentrations used in the univariate analysis were used as input, and pathway perturbances between infected and control samples were considered. The *Escherichia coli* pathway library was selected for the analysis. The algorithms used for enrichment analysis and pathway topology analysis were the global test and relative betweenness centrality, respectively. Pathways were considered significant when the *P*-values calculated from the enrichment analysis were less than 0.05, and the pathway impact scores were greater than 0.05.

### Metabolite quantification

#### Fluoride

Samples were diluted at least 1:100 and 25 µL injected onto a ICS5000 two-dimensional ion chromatography system (Thermo Scientific). This used a 4-mm AS19 anion exchange column in the first-dimension running water and potassium hydroxide mobile phases at 1 mL/min, using a gradient from 8 mM to 10 mM KOH over 25 min before column washing and re-equilibration. The second dimension consisted of a MAC-200 anion trapping column followed by a capillary AS19 anion exchange column using water and KOH mobile phases ran at 0.1 mL/min. Under these first dimensional chromatographic conditions fluoride eluted at 6.6 min. For a cut window from 6.25 to 7.15 min the eluent was transferred to the trapping column and then to the capillary column. The capillary column was run isocratically at 9 mM KOH for the first 8 min and then from 9 to 10.1 mM KOH from 8 to 30 min, before column washing and re-equilibration. Fluoride in the samples was quantified against a standard curve using a seven-anion ion chromatography standard containing fluoride (Thermo Scientific) and multiplied by the dilution factor of the sample.

L-Lactate and D-lactate were quantified using enzymatic assays following the manufacturer’s instructions (L-LATE and K-LATE, Megazyme, Ireland).

### Mouse UTI model

The C57BL/6 mouse model of ascending UTI was employed as previously described (24, 73). Strains were enriched for type 1 fimbriae expression by three successive rounds of static growth in LB without salt for 48 h followed by one round of static growth for 24 h for inoculum preparation and did not display any difference in type 1 fimbriae production as assessed by yeast cell agglutination and a fim switch orientation PCR. Infections were performed as competitive assays; the inoculum contained a 1:1 strain mixture of EC958∆*lac* (WT) versus mutants. Bacterial loads corresponding to each strain in the urine and bladder at 24 h post infection were enumerated by plating onto MacConkey agar. A competitive index was determined as the ratio of each respective mutant versus WT to the ratio of the two strains in the inoculum. Statistical analyses were performed using the two-tailed Wilcoxon signed-rank test (Prism7, GraphPad).

## Data Availability

TraDIS sequencing data are available under the BioProject ID PRJNA1044227 on NCBI. NMR data were deposited in the MetaboLights database (74, 75) under accession number MTBLS9131.

## SUPPLEMENTAL MATERIAL

Figure S1 mbio.03388-23-s0001.tiff Generation times of reference UPEC strains compared to MG1655.

Figure S2 mbio.03388-23-s0002.tiff Genes required for growth in HU identified by TraDIS.

Figure S3 mbio.03388-23-s0003.tiff Comparison of growth curve parameters.

Figure S4 mbio.03388-23-s0004.tiff Prevalence and sequence similarity of genes identified in this study to be required for growth in HU.

Figure S5 mbio.03388-23-s0005.tiff Pathway analysis of the metabolic pathways altered following growth of EC958 in HU.

Figure S6 mbio.03388-23-s0006.tiff Genes identified in this study are highly expressed in urine of UTI patients.

Supplemental file mbio.03388-23-s0007.docx Supplemental figure and table legends.

Supplemental Tables mbio.03388-23-s0008.xlsx Tables S1-S6.

ASM does not own the copyrights to Supplemental Material that may be linked to, or accessed through, an article. The authors have granted ASM a non-exclusive, world-wide license to publish the Supplemental Material files. Please contact the corresponding author directly for reuse.
