# Supplementary figures and images for: Combined functional genomic and metabolomic approaches identify new genes required for growth in human urine by multidrug-resistant Escherichia coli ST131
Source: mBio. 2024 Feb 14;15(3):e03388-23. doi: 10.1128/mbio.03388-23 (PMC10936160; doi:10.1128/mbio.03388-23)

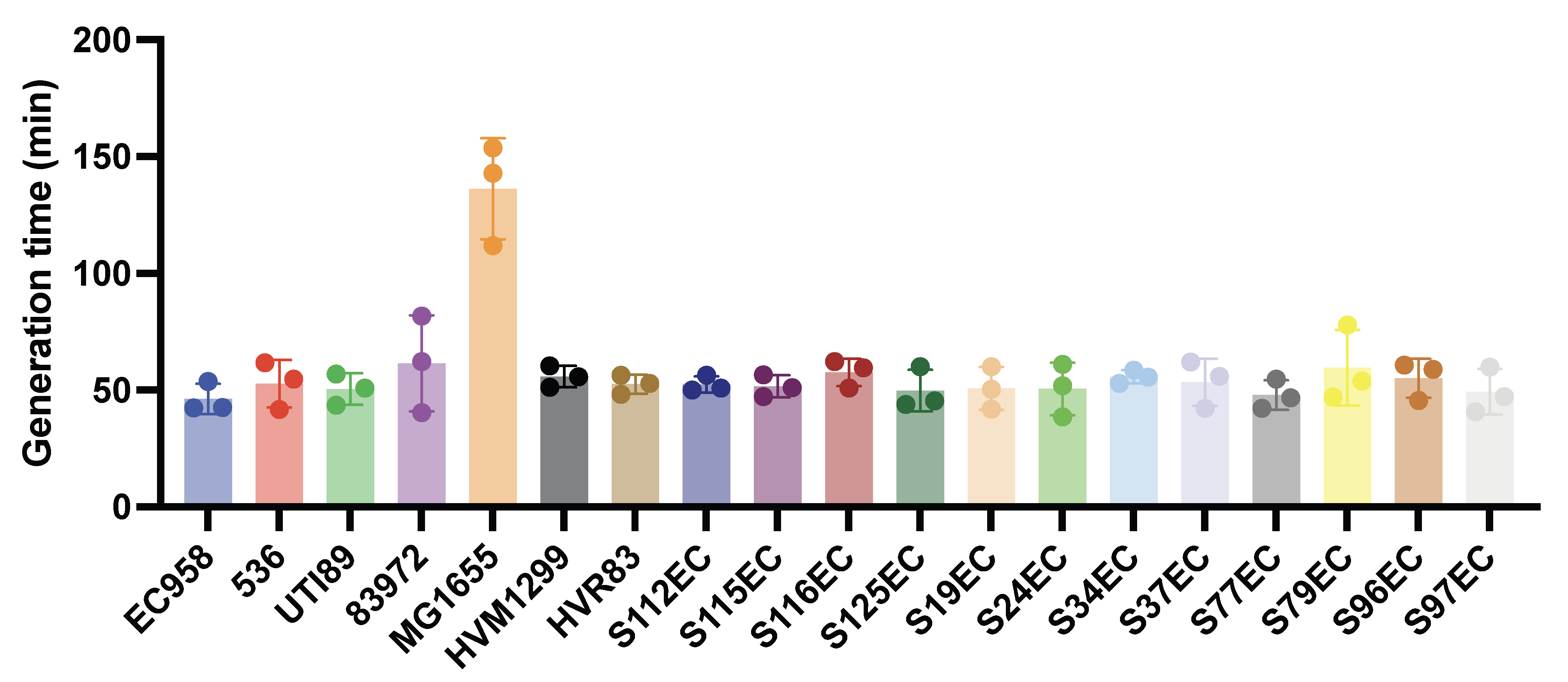

Supplement: Figure S1 — Generation times of reference UPEC strains compared to MG1655. [file mbio.03388-23-s0001.tiff]

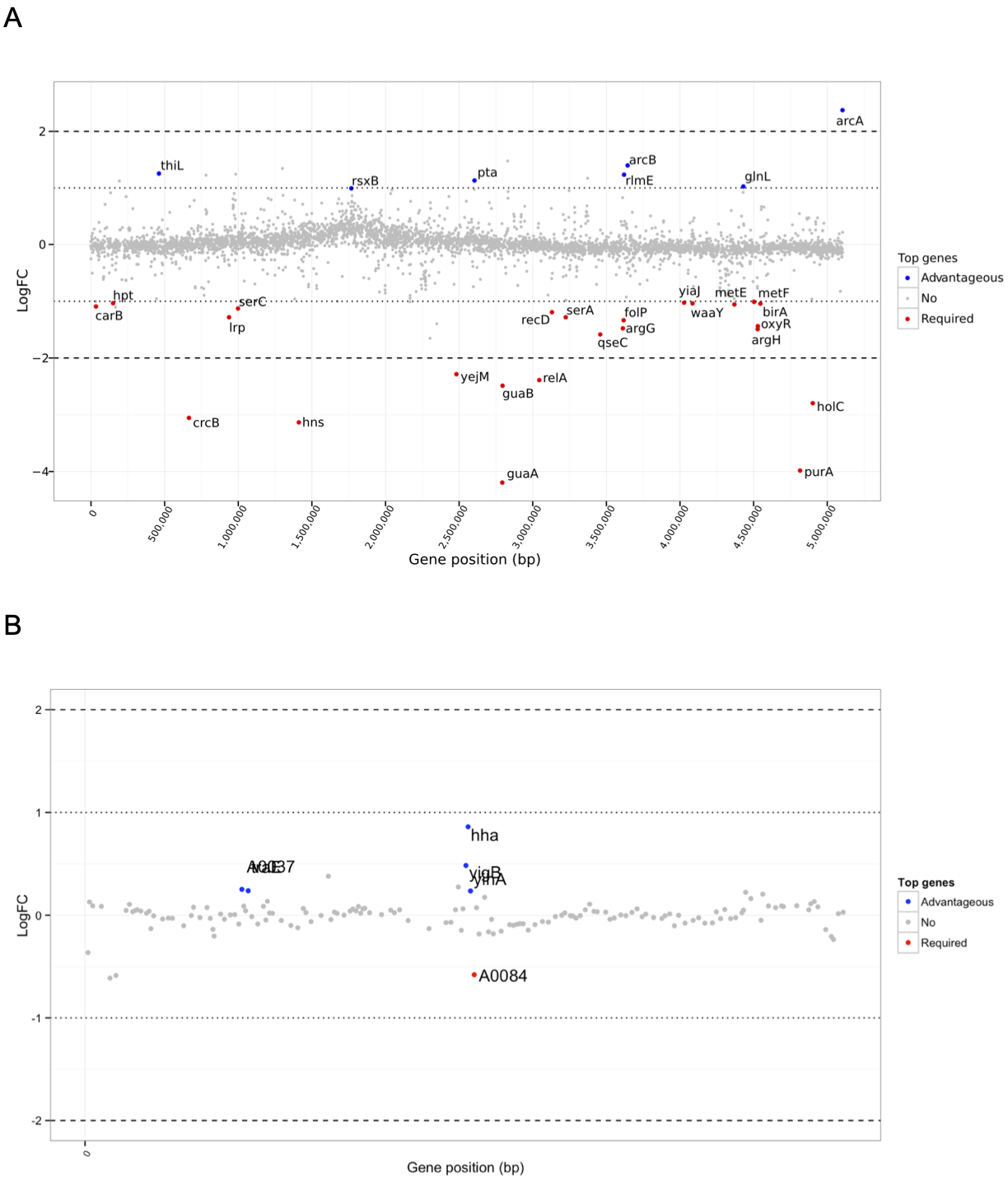

Supplement: Figure S2 — Genes required for growth in HU identified by TraDIS. [file mbio.03388-23-s0002.tiff]

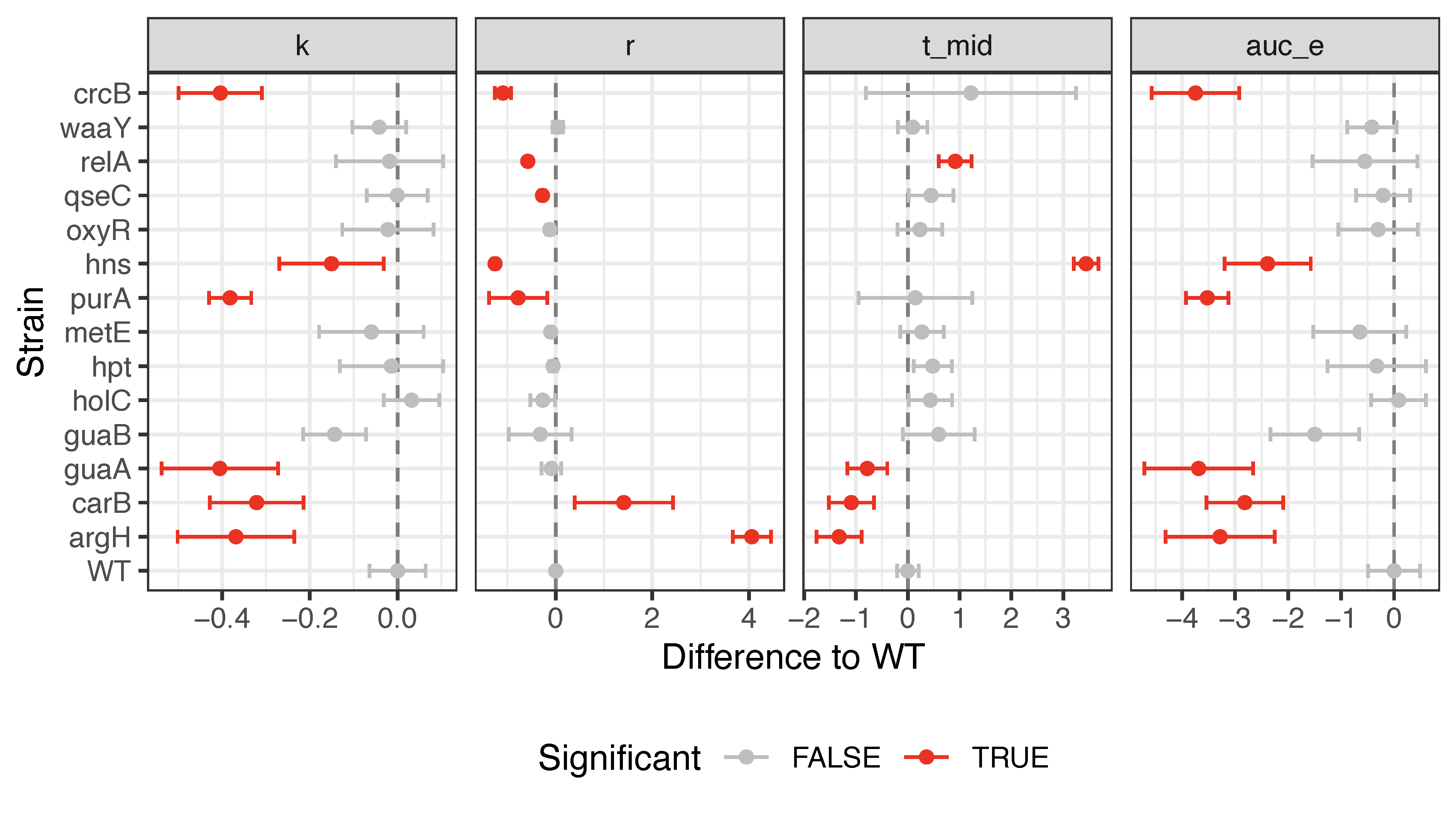

Supplement: Figure S3 — Comparison of growth curve parameters. [file mbio.03388-23-s0003.tiff]

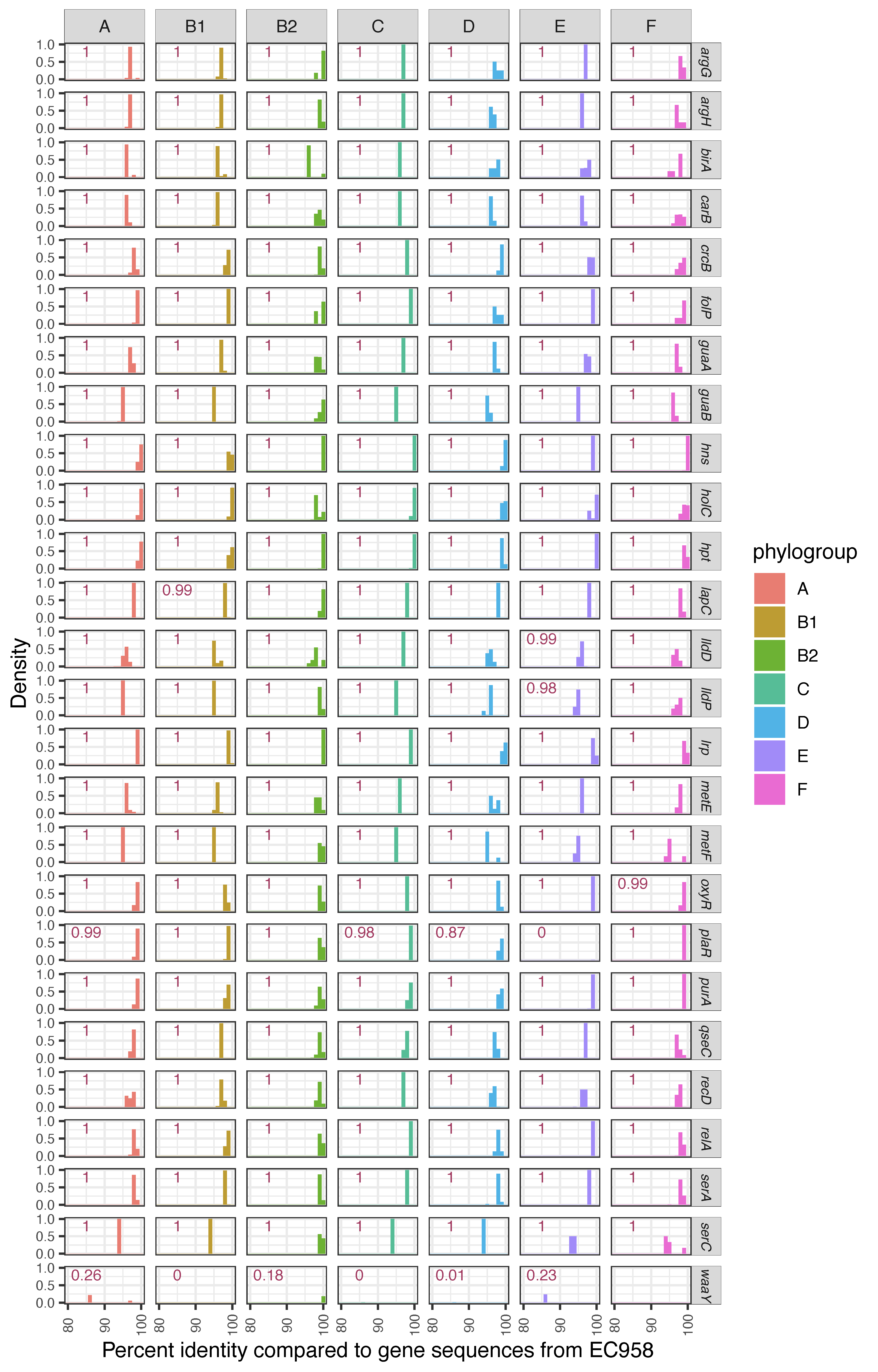

Supplement: Figure S4 — Prevalence and sequence similarity of genes identified in this study to be required for growth in HU. [file mbio.03388-23-s0004.tiff]

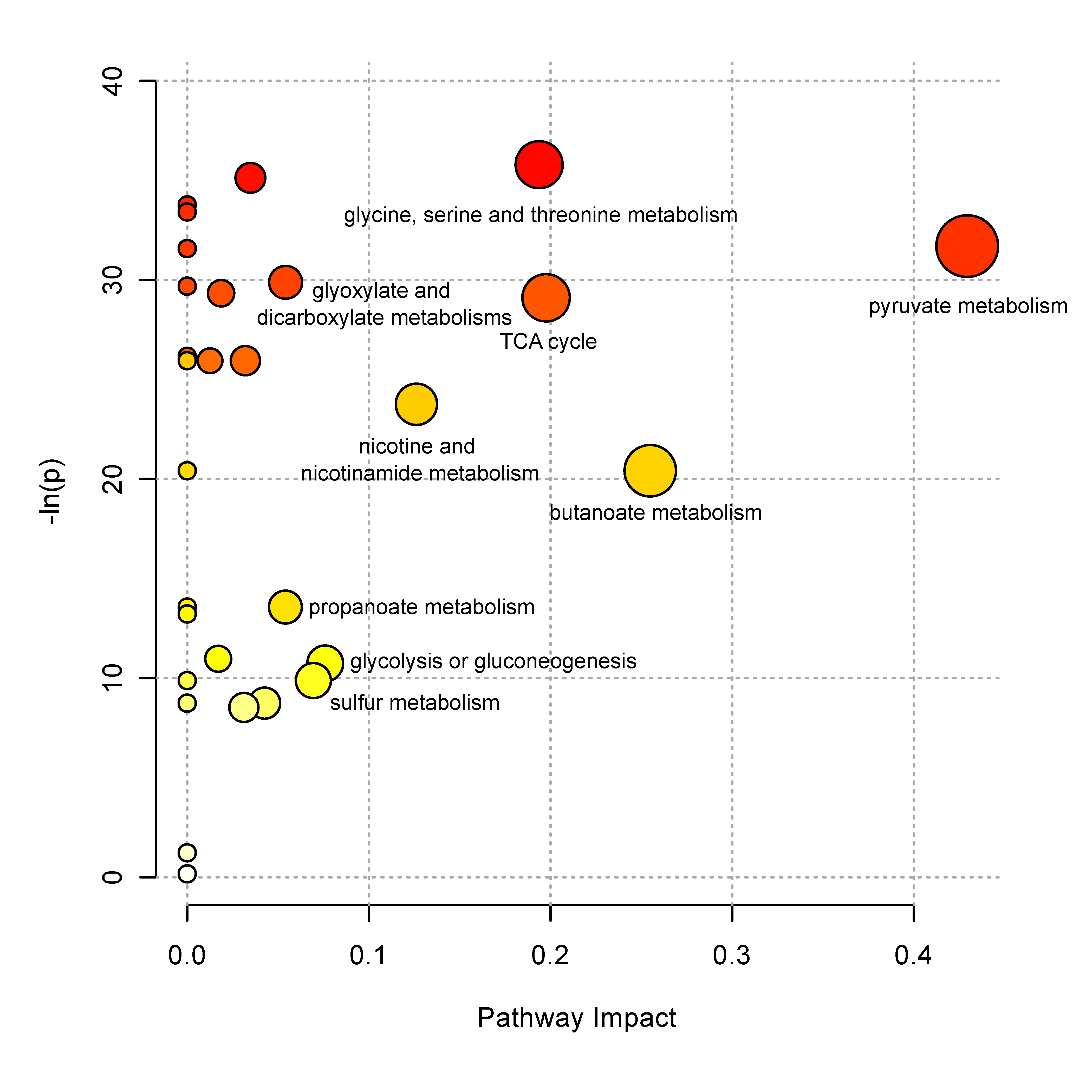

Supplement: Figure S5 — Pathway analysis of the metabolic pathways altered following growth of EC958 in HU. [file mbio.03388-23-s0005.tiff]
