# Supplementary material for: Combined functional genomic and metabolomic approaches identify new genes required for growth in human urine by multidrug-resistant Escherichia coli ST131
Source: mBio. 2024 Feb 14;15(3):e03388-23. doi: 10.1128/mbio.03388-23 (PMC10936160; doi:10.1128/mbio.03388-23)
Supplement: Supplemental file — Supplemental figure and table legends. [file mbio.03388-23-s0007.docx]

**Supplementary Tables (in excel file)**

**Table S1** Summary of sequencing and mapping results of multiplexed TraDIS runs.

**Table S2** Results of TraDIS analysis comparing Test vs Control samples for all genes in EC958 chromosome.

**Table S3** Pathways containing gene(s) that was identified by TraDIS.

**Table S4** Concentrations of Fluoride, L-Lactate and D-Lactate in HU used in this study.

**Table S5** List of metabolite changes in HU before and after UPEC growth.

**Table S6** List of primers used in this study.

**Supplementary Figures**

**FIG S1**. Generation times of reference UPEC strains compared to MG1655. Generation times were calculated from time range of 1-2h of the growth curves. Shown is the mean and all data points from 3 independent experiments.

**FIG S2. Gene required for growth in HU identified by TraDIS.** The plots show the genomic position and log_2_ Fold Change (LogFC) of read counts between growth in LB versus growth in HU. (A) Genes on the chromosome and the red dots indicate genes that were determined as required for growth in HU. (B) Genes on the pEC958 plasmid; blue or red dots indicate FDR < 0.001 but no genes on the plasmid pass our LogFC cut off to be determined as required for growth in HU.

**FIG S3. Comparison of growth curve parameters.** Parameters were estimated by the R package growthcurver (version 0.3.1) including ‘k’ (maximum possible population size, or carrying capacity), ‘r’ (growth rate), ‘t_mid’ (the time at which the population density reaches ½k), and ‘auc_e’ (empirical area under the curve). For each parameter in each mutant strain, a two-sample t-test was performed comparing the mutant and the WT. The estimates of mean difference between mutant and WT were plotted as dots with 95% confident interval indicated by error bars. The p-values were adjusted by Benjamini and Hochberg method to control for false discovery rate due to multiple comparisons. Adjusted p-values ≤ 0.05 were shown in red.

**FIG S4. Prevalence and sequence similarity of genes identified in this study to be required for growth in HU.** Each individual graph shows a histogram of the distribution of percent identity of a gene from EC958 compared to the respective gene found in genomes belong to each phylogroup. Percent identities were obtained by blastn of EC958 genes against a database of 100 genomes randomly selected from the top 100 STs in *E. coli* from Enterobase (downloaded on 18/12/2020). The number in each individual graph indicates the proportion of genomes that contain the respective gene (i.e. 1 = 100% of genomes contain the gene). The threshold to determine whether a gene is present in a genome is percent identity ≥ 80% and alignment length ≥ 80%. The *waaY* gene displayed a reduced prevalence in the genomes of all phylogroups investigated, and to a lesser extent the *plaR* gene in phylogroup D and E. The number of genomes in each phylogroup: A, 2968; B1, 3700; B2, 1100; C, 400; D, 800; E, 400; F, 600.

**FIG S5**. **Pathway analysis of the metabolic pathways altered following growth of EC958 in HU**. The analysis was performed using the *E. coli* pathway library in MetaboAnalyst 4.0 (71, 72). The pathways are represented as circles according to their scores from enrichment (vertical axis) and topology analysis (pathway impact, horizontal axis). Darker circle colors indicate more significant changes of metabolites in the corresponding pathway. The size of the circle corresponds to the pathway impact score and is correlated with the centrality of the involved metabolites. Pathways were annotated when the *P*-values calculated from the enrichment analysis were <0.05 and the pathway impact score was >0.05.

**FIG S6. Genes identified in this study are highly expressed in urine of UTI patients.** (A) Expression levels of genes identified in this study obtained from RNA-seq data of 21 *E. coli* strains in the urine of elderly patients suffering from an acute UTI (18). The dotted line indicates the mean expression levels of all genes in all samples in this dataset (excluding genes with nRPK = 0 in all samples). nRPK: number of reads per kilobase. (B) The increase in expression levels of L-lactate dehydrogenase (*lldD*) in LB media compared to healthy human urine (HU) and human urine suffering from acute UTI (UTIU). Data extracted from (17). Statistical analysis was performed using Kruskal-Wallis test with Dunn’s multiple comparison test.
